# Catalytic Transesterification of Cellulose Nanocrystals (CNCs) with Waste Oils: A Sustainable and Efficient Route to Form Reinforced Biofilms

**DOI:** 10.3390/polym17212877

**Published:** 2025-10-28

**Authors:** Antonio De Nino, Antonio Jiritano, Federica Meringolo, Paola Costanzo, Vincenzo Algieri, Enrica Fontananova, Loredana Maiuolo

**Affiliations:** 1 Department of Chemistry and Chemical Technologies, University of Calabria, Via P. Bucci, Cubo 12C, 87036 Rende, CS, Italy; denino@unical.it (A.D.N.); federica.meringolo@unical.it (F.M.); paola.costanzo@unical.it (P.C.); 2 IRCCS NEUROMED—Istituto Neurologico Mediterraneo, Via Atinense 18, 86077 Pozzilli, IS, Italy; vincenzo.algieri@unical.it; 3 Institute on Membrane Technology of the National Research Council (ITM-CNR), University of Calabria, Via P. Bucci, Cubo 17/C, 87036 Rende, CS, Italy; e.fontananova@itm.cnr.it

**Keywords:** biomaterials, cellulose, exhausted oils

## Abstract

Bioplastics are plastics derived from natural resources like corn starch, biomass, sugarcane bagasse, and food waste. Unlike fossil-fuel-based plastics, they are entirely or partially bio-degradable. Cellulose- and starch-based bioplastics are already used for applications like packaging, cutlery, bowls, straws, and shopping bags. With the aim of developing eco-friendly biofilms for various applications, cellulose nanocrystals (CNCs) were obtained by sulfuric acid hydrolysis of waste cellulose and functionalized by transesterification with exhausted oils. The resulting transesterified nanocellulose (TCNC) was used as a reinforced material of PLA at different concentrations to develop biofilms using the solvent casting method. The biofilms composed of PLA and TCNC were assessed through Fourier-transform infrared spectroscopy (FTIR), mechanical properties, moisture barrier property (water vapor permeability rate—WVTR), and measurements of the water contact angle (WCA). A scanning electron microscopy (SEM) analysis confirmed the high compatibility of the PLA blended with TCNC at 1% and 3%. The inclusion of transesterified cellulose nanocrystals (TCNCs) to PLA increased the hydrophobicity, the film tensile strength, and the water vapor barrier properties of the final composite films.

## 1. Introduction

The widespread and continuous demand for new sustainable materials utilizing eco-friendly resources stems from the imminent depletion of the petroleum-derived resource and significant environmental issues regarding to waste disposal, and pollution [1]. For example, there is a large amount of food waste, including fruits (i.e., orange peel), vegetables, and forest residues, and many routes are taken to recycle this waste. Among them, one of the most important processes is the extraction of cellulose (CE) from these residue materials to form new cellulose-based biopolymers [2,3], considered as an environmentally friendly alternative to fossil resources, especially for polymer chemistry [4,5,6,7]. Unfortunately, despite its many benefits (i.e., renewability, biodegradability, biocompatibility, non-toxicity, low cost, and thermal and chemical stability), the highly polar nature of cellulose due to its OH-rich structure poses difficulties to its dissolution in common organic solvents and water, as well as poor processability and chemical modification [8]. Therefore, in order to confer better physical–chemical properties to cellulose, such as solubility, water resistance, durability, and malleability, cheap and sustainable chemical modifications are needed to make the cellulose-based materials competitive against traditional plastics without destroying its many intrinsic properties. This strategy proved to be effective in improving the mechanical performances of fairly apolar materials using cellulose-based waste [9]. In particular, the esterification reactions of the cellulose hydroxyls by inserting short [10,11] and long chains to produce less hydrophilic ester groups can be considered an efficient way to turn the cellulose surface into a more hydrophobic one [12,13,14].

Waste cooking oil (WCO) is a renewable resource that can be obtained from food processing industries, restaurants, and other sources that use a large amount of oil, and its reuse can effectively solve the problem of waste oil pollution [15]. Vegetable oils are abundant and renewable sources of triglycerides, and, for this reason, WCO has received considerable attention for its potential utilization in rendering hydrophobic cellulose fibers as a high-value-added application to produce bio-based composite materials [16]. The waste origin might influence the quality and amount of triglyceride. In particular, the lipophilic moieties in vegetable oils include palmitic (C16:0), stearic (C18:0), oleic (C18:1), linoleic (C18:2), and linolenic (C18:3) chains.

Chemical modifications of the OH groups of cellulose may represent an interesting method to introduce solubility and processability to a recalcitrant material as the pristine cellulose. The transesterification reaction is widely known commercially in industrial organic reactions and consists of the conversion of an ester into another ester. Therefore, the transesterification reaction between triglycerides and cellulose hydroxyls may form a hydrophobic surface on cellulose due to the long acyl chains. However, to our knowledge, it is not easy to perform this reaction on cellulose due to its recalcitrant nature, and only one complex procedure is reported in the literature [17].

Between the potential chemical strategies, the heterogeneous modification of cellulose is widely employed for the synthesis of cellulose derivatives, considering that its pre-functionalization is not a requirement unlike in homogeneous reactions [18]. Orange peel waste (OPW) is a promising byproduct among whole vegetable discards, considering that orange fruits is one of most produced fruits worldwide [19,20]. More in detail, cellulose fibrils from orange peel waste have a relatively low crystallinity index (CI, 58%) and high colloidal stability that make them a material ideal for subsequent chemical transformations such as the transesterification [21].

In the ongoing search for novel biomaterials as an alternative to conventional plastics, polylactic acid (PLA) is one of the most representative of the biodegradable, readily available, and abundant biopolymers [22]. We chose to focus on this material because of its widespread use, as a consequence of its undiscussed properties. It is highly transparent, elastic, and biodegradable (i.e., well fit for composting), which are appealing properties for disposable products development [23]. In addition, it is quite versatile from the chemical point of view, having proved to possess quite a good affinity with other polymers and OH-rich materials [24]. However, the inherent brittleness of PLA makes it subject to low tensile strength, which can be improved by adding functionalized cellulose fibers, even if the addition of pristine cellulose to PLA produces biofilms with preferential breaking points. This effect is surely due to the highly poor homogeneity of the two materials in the manufacturing process.

The goal of this work was to prepare a novel and cheap composite biomaterial applicable as films with an elevated performance (i.e., high mechanical resistance, and great biodegradability) by adding a functionalized biomaterial to PLA biofilms. More in detail, this derivatized biomaterial was realized by a transesterification reaction between the wastes of nanocellulose crystals (CNC) resulting from citrus-processing by-products (i.e., orange peels) and exhaust oils deriving from cooking food. This modification permits the introduction of fatty acid chains onto the nanocellulose structure, creating a hydrophobic coating and enhancing the compatibility with lipophilic polymers as PLA. In particular, we decided to use exhausted sunflower oil with a high oleic acid content (HOSO, High Oleic Sunflower Oil with oleic acid > 80%) that nowadays is highly employed for frying and baking, considering its greater stability at high temperatures than traditional sunflower oil. The strong point of our method relies on a simple experimental procedure, considering the use of waste reagents, the absence of a solvent, the open-system reaction conditions, the relatively low temperatures, the reduced reaction time, and the use of an α-hydroxyacid as a catalyst, necessary for the formation of an intermediate that acts as an acylating agent. In particular, the use of an α-hydroxyacid avoids the addition of any activating agents. In this regard, we have proposed a mechanism of the transesterification reaction.

Then, we incorporated the transesterified nanocellulose (TCNC) into PLA matrices to produce composite biofilms, which were evaluated for mechanical and barrier properties, with the aim of reducing the costs of disposal of waste deriving from previous processes, and improving the mechanical characteristics of the obtained composite films for possible applications in many fields, including packaging and shopping bag production [25,26].

## 2. Materials and Methods

### 2.1. Chemicals

Cellulose derived from citrus waste was furnished by JRS Silvateam Ingredients S.r.l. (Rende, CS, Italy). PLA pellets were furnished by Delta Plast S.r.l. (Figline Vegliaturo, CS, Italy). Exhausted sunflower oil HOSO was furnished by various local catering businesses. l-(+)-tartaric acid was purchased from Merck Life Science S.r.l. (Milano, MI, Italy) at 99% purity grade. Methanol and chloroform were purchased from Honeywell at a high purity grade and used without further purification. Acetone, sodium hydroxide, and sulfuric acid were purchased from Merck Life Science S.r.l. (Milano, MI, Italy) at a high purity grade and used without further purification.

### 2.2. Preparation of Nanocellulose from Orange Peel Waste Cellulose

Cellulose nanocrystals (CNCs) were obtained by sulfuric acid hydrolysis of orange peel waste cellulose. Before hydrolysis, cellulose was soaked in 90% aqueous acetone for 24 h to remove all the extractives. The obtained mixture was washed by water until it reached a neutral pH. Subsequently, cellulose was hydrolyzed with 64% (*w*/*w*) sulfuric acid solution at 45 °C for 30 min with constant stirring. Then, the suspension was diluted 20-fold in distilled water to quench the reaction. After that, the suspension was centrifuged at 6000 rpm for 10 min to concentrate the obtained nanocellulose (NC) and to remove excess aqueous acid. The resultant precipitate was filtrated and washed with distilled water until it reached a neutral pH. With the aim of improving thermal stability of prepared CNC, distilled water was added to isolated nanocellulose and resulting suspension was neutralized with the addition of sodium hydroxide (NaOH) 0.25 M. Finally, CNC was washed several times with distilled water to eliminate the excess sodium hydroxide. Colloidal nanocellulose was achieved by sonication at 40% power output for 5 min.

CNC weight concentration within the colloidal suspension obtained by sonication has been estimated according to the following Equation (1):

(1) $$\rho_{solution}=\frac{1}{\left(\frac{w_{H_{2}O}}{\rho_{H_{2}O}}+\frac{w_{CNC}}{\rho_{CNC}}\right)}$$

where $w_{H_{2}O}$ and $w_{CNC}$ are the weight fractions of water and cellulose nanocrystals, respectively, and $\rho_{H_{2}O}$ and $\rho_{CNC}$ are the densities of raw water (1 g/cm^3^) and cellulose nanocrystals taken as 1.676 g/cm^3^ [27]. According to this equation, CNC concentration was found to be 2 wt%.

### 2.3. Transesterification of CNC with Exhausted Sunflower Oil (HOSO)

CNC **1** (1 g), tartaric acid (1.8 g), and exhausted sunflower oil **2** (HOSO, 20 g) were placed in a round-bottom flask equipped with a bubble condenser and heated to 120 °C under stirring for 6 h. Then, the mixture was allowed to cool down to room temperature, and the solid product was separated by vacuum filtration. Several washings of the recovered solid by hot methanol were performed to guarantee the removal of the catalyst and the excess sunflower oil. A further purification process, Soxhlet extraction by methanol, was conducted to ensure that the excess oil was removed, and then the obtained product was dried in an oven at 70 °C for 24 h, recovering the transesterified cellulose (TCNC, **3**) as a pale-yellow solid.

### 2.4. Synthesis of Diacetyl Tartaric Acid (***7***)

A solution of l-(+)-tartaric acid **4** (2 g, 13.3 mmols) in acetyl chloride **6** (20 mL, 280 mmols) was kept under magnetic stirring at reflux temperature for 48 h. After this period, the reaction mixture was evaporated, and the solid residue obtained was recrystallized in AcOEt/Hexane 1:1, providing the compound **7** as a white solid. Compound **7** was characterized by FT-IR, ^1^H NMR, ^13^C NMR, and COSY NMR analysis (see Supplementary Materials).

### 2.5. Preparation of Composite Films Between TCNC or CNC with PLA by Solvent Casting Method

PLA (1 g) was dissolved in chloroform (15 mL) in a beaker and a specific amount of additive (TCNC or CNC, 1%, 3%, 5%, or 10% in weight with respect to polymer) was dispersed in 5 mL of chloroform under stirring. After 30 min, the two solutions were mixed and left to be stirred for another 30 min. After this time, the final blend of PLA and additive was poured into an opportune glass container, and the solvent was allowed to evaporate, obtaining the final film. The film was gently peeled off the glass container and cut to size for mechanical and permeability tests.

### 2.6. FT-IR and SEM Characterization

FT-IR spectra (KBr pellets) were recorded with a Jasco FT/IR-4200 spectrometer (JASCO EUROPE s.r.l.—Cremella, LC, Italy) in a frequency range between 450 and 4000 cm^−1^. For IR spectra of CNC (**1**), sunflower oil HOSO (**2**), TCNC (**3**), diacetyl tartaric acid (**7**), and acetylated CNC (**8**), see Supplementary Materials.

Morphological studies were carried out using a Phenom ProX (Thermo Fisher Scientific—Phenom-World B.V., Eindhoven, The Netherlands) scanning electron microscope (SEM), operating under vacuum conditions of 8 × 10^−6^ Torr at an accelerating voltage of 15 kV.

### 2.7. NMR Characterization of TCNC (***3***) and Diacetyl Tartaric Acid (***7***)

^1^H NMR experiment for **3** was recorded at 500 MHz, in DMSO-*d*_6_ as solvent using tetramethylsilane (TMS) as an internal standard and at 37 °C (Bruker Avance 500 MHz with a 5 mm TBO probe, Rheinstetten, Germany). ^1^H, COSY, and ^13^C NMR experiments for **7** were recorded at 500 and 125.7 MHz, respectively, in CDCl_3_ as solvent using tetramethylsilane (TMS) as an internal standard (Bruker Avance 500 MHz with a 5 mm TBO probe, Rheinstetten, Germany). Chemical shifts are given in parts per million and coupling constants in Hertz.

### 2.8. Water Vapor Transmission Rate

The water vapor transmission rate was measured following the ASTM E96-95 gravimetric method [28]. The cell employed to measure the permeability is a small container made of nylon, which is partially filled with water and a polymeric membrane at the top. The measurements were carried out at 25 °C in a balance and the mass loss was recorded. All experiments were performed in triplicate. All data are expressed as the mean ± standard deviation (see Supplementary Materials).

### 2.9. Mechanical Tests

Mechanical tests were performed in accordance with ISO 527-1:2012 procedure [29] employing an electronic dynamometer (Acquati) and tests were performed at “Delta Plast s.r.l.” (Figline Vegliaturo, CS, Italy).

### 2.10. Water Contact Angle (WCA) Measurements

The composite film surface wettability was evaluated by static water contact angle (WCA) measurements carried out at room temperature using a CAM 200 device (KSV Instruments, Ltd., Helsinki, Finland) using Young’s wetting model to determine the equilibrium contact angle. On the surface of each composite film, a 5 µL drop of distilled water was deposited by a microsyringe and an automatic dispenser. The angle between the surface tangent on the water–air interface and the tangent on the film–water interface at their intersection was measured from 5 video-captured images taken at 40 ms intervals immediately after drop deposition. Moreover, 5 additional images were taken at 1 s intervals to assess that an equilibrium state was reached. Three sequential measurements were averaged for each sample, depositing three drops in three different points of each surface, and the standard deviation was calculated for each experiment.

## 3. Results and Discussion

### 3.1. Preparation and Transesterification of CNC

Initially, with the aim of using the transesterified cellulose nanocrystals (TCNCs) as an additive in the formation of bio-composites with PLA, we prepared cellulose nanocrystals (CNCs) that were obtained by the sulphuric acid hydrolysis of waste cellulose deriving from orange peels, according to a procedure described in the literature [30].

**Scheme 1:**
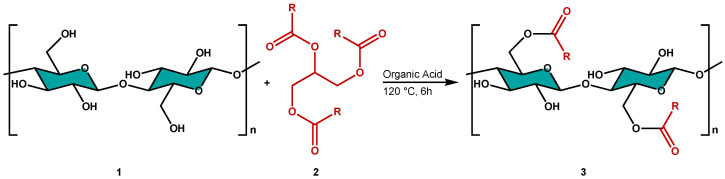
Transesterification reaction between cellulose and triglycerides of sunflower oil.

Then, we performed the transesterification reaction of CNCs with an exhausted sunflower oil (HOSO) as an acylating agent. The esterification reaction was catalyzed by an α-hydroxyacid in the heterogeneous phase, as illustrated in Scheme 1.

The reaction progress was controlled based on the percentage of grafting of the initial cellulose with the waste sunflower oil according to the following Formula (2):

(2) $$P_{G}=\frac{m_{2}-m_{1}}{m_{1}}\times 100$$

where m_1_ is the mass of the initial cellulose and m_2_ is the mass of the functionalized cellulose. The reaction was optimized by varying different parameters such as the time, temperature, and organic acid used as a catalyst. The optimization of the reaction conditions is reported in Table 1.

**Figure 1:**
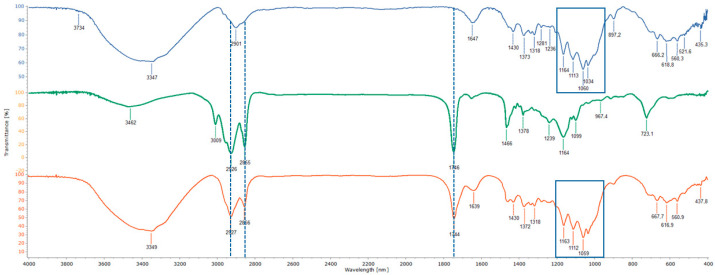
FT-IR spectra of CNC (blue), exhausted sunflower oil (green), and TCNC (red).

From the table data, it is possible to observe how the maximum grafting was obtained in entry 4, even if similar grafting values were observed in entries 2 and 3. For this reason, we decided to consider the optimal reaction conditions as reported in entry 2, considering a minor reaction time. Subsequent attempts by increasing the temperature did not lead to a greater yield, probably because of the decomposition phenomena (entries 5 and 6). Moreover, we tried to change the organic acid, and we observed a minor grafting of the reaction with malic and succinic acid (entries 7 and 8). Meanwhile, in the presence of adipic or oxalic acid (entries 9 and 10), it was not possible to observe any grafting. Lastly, the reaction was also performed in the absence of the organic acid (entry 11), and, as can be seen, a complete absence of the final product was observed, confirming that the reaction route needs catalysis. Therefore, the best reaction conditions were found by using tartaric acid as an organic catalyst, a temperature of 120 °C, and 6 h as the reaction time. TCNC was confirmed by FT-IR spectroscopy, as shown in Figure 1, in which the IR spectra of functionalized nanocellulose (TCNC), exhausted sunflower oil, and the starting nanocellulose crystals (CNCs) are reported.

It is worth noting the presence of a broad band at the very high side frequency in the 3750–3100 cm^−1^ range (in CNC and TCNC spectra). This feature is generally due to the OH functional groups in bound parts of molecules (interacting functional groups). The absence of sharp contributions at the high-frequency side emerging from the broad band rules out the presence of some monomers (not interacting OH^−^ containing molecules), confirming the polymeric nature of the materials in the bulk state.

In the 3000–2800 cm^−1^ spectral range, the peaks due to the symmetric and antisymmetric stretching (ν_S_ and ν_AS_, respectively) of CH_2_ and CH_3_ groups are visible. The different spectrum profile in the three samples is due to the fact that the relative intensity ratio of such peaks is sensitive to the molecular lateral packing of the alkyl chains, as found in some model systems [31]. Coherently, the bands at ~1455 cm^−1^ and ~1375 cm^−1^, due to the deformation vibrations of CH_2_/CH_3_ and CH_3_, respectively, are present at different positions for the three samples. The appearance of a peak at 1242 cm^−1^ is associated with C–O–C vibrations from ester groups and is clearly visible in the spectrum of waste oil, corresponding to its stretching vibration of C–OH bonds.

Importantly, in the spectrum of TCNC, it is possible to observe the characteristic signals of cellulose at 1220–960 cm^−1^ (see purple boxes) [32] and those of exhausted sunflower oil (aliphatic stretching at 3050–2800 cm^−1^ and carbonyl stretching at 1744 cm^−1^; see dotted black lines) [33].

**Figure 2:**
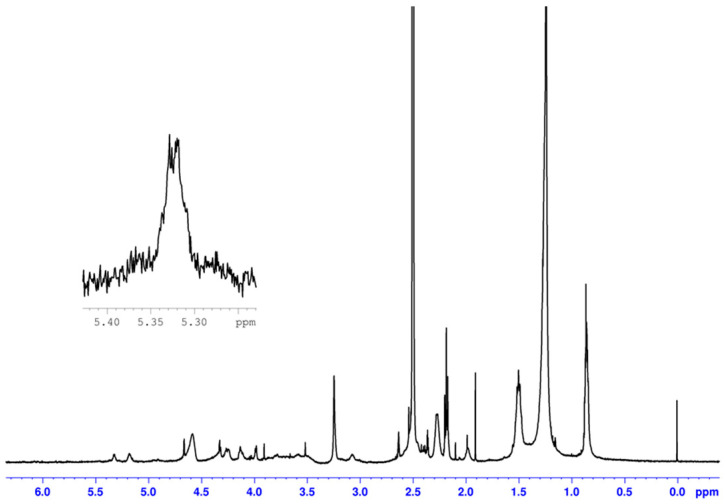
^1^H NMR of TCNC.

In addition, the structure of TCNC was confirmed by ^1^H NMR spectroscopy as reported in Figure 2.

All signals were attributed in analogy with those reported in reference [17]. As can be seen, the protons on unsaturated carbons of the oleic chain are observed at a chemical shift of 5.33 ppm (see enlarged signal), while the peaks between 5.24 and 3.00 ppm can be attributed to the protons of the cellulose backbone. Furthermore, the peak at 1.24 ppm can be assigned to methylene groups of the oleic aliphatic chain (see Supplementary Materials for ^1^H NMR spectrum).

### 3.2. Mechanism Hypothesis of Transesterification Reaction

**Scheme 2:**
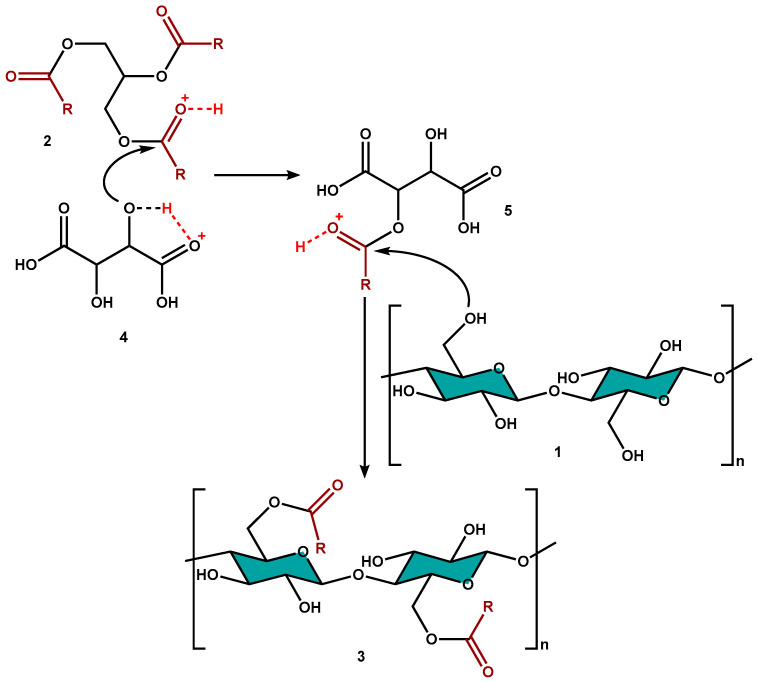
Proposed mechanism of transesterification reaction.

Considering the absence of literature data about the mechanism of the transesterification between sunflower oil and CNC, and to better understand how this reaction occurs, a reaction mechanism has been hypothesized, in analogy to the esterification of bacterial nanocellulose (BNC) by short-chain carboxylic acids reported in literature (Scheme 2) [34]. More in detail, it is possible to hypothesize an intermolecular protonation of the carbonyl of the triglyceride **2** by tartaric acid **4** and a contemporary intramolecular sharing of the hydroxyl proton of the α-hydroxy acid with its carboxyl group. The resulting reduction in the energy gap HOMO-LUMO favors the intermediate formation **5** that undergoes nucleophilic attack by the primary hydroxyl groups of cellulose **1**, providing the final esterified product **3**.

**Scheme 3:**
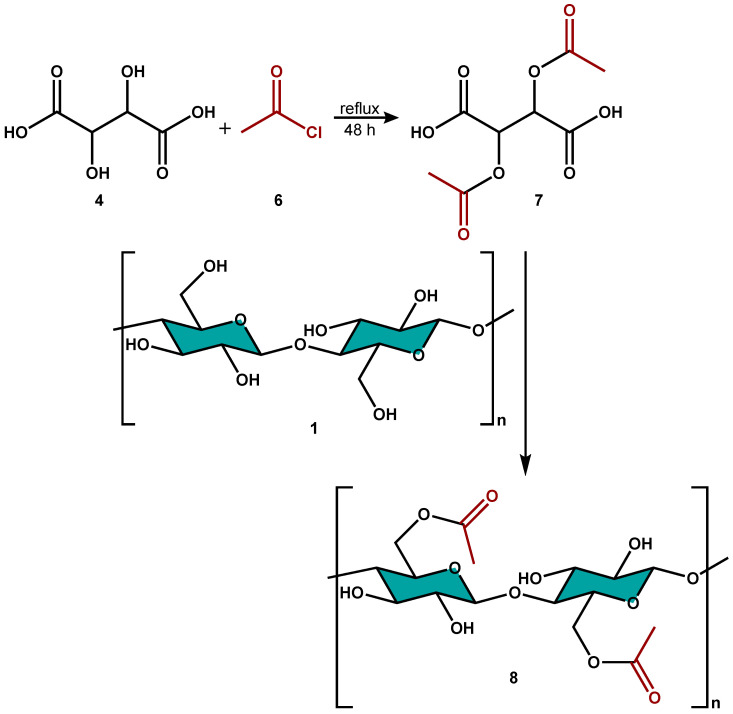
Formation of diacetyl tartaric acid and subsequent acetylation of CNC.

To support this hypothesis, we simulated the intermediate **5** of Scheme 2 through the preparation of the diacetyl tartaric acid **7** (Scheme 3), by using acetyl chloride **6** as the acylating agent and tartaric acid **4**. The acetylated intermediate **7**, isolated and characterized by FT-IR, ^1^H NMR, ^13^C NMR, and COSY NMR analysis (see Supplementary Materials), represented the key intermediate for the proposed mechanism when it reacted with CNC **1** to produce the acetylated CNC **8** (Scheme 3), as confirmed by FT-IR analysis (see Supplementary Materials).

### 3.3. Morphological Analysis and Hydrophobicity Tests of TCNC

**Figure 3:**
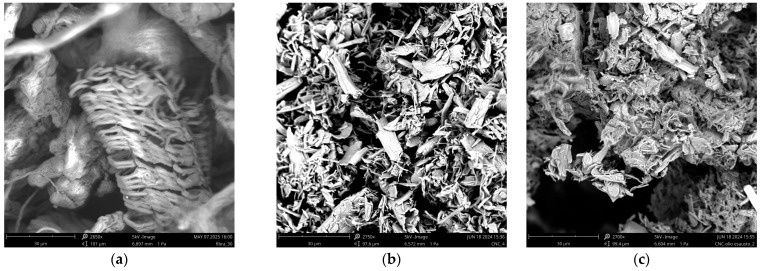
SEM images of (**a**) cellulose waste deriving from orange peels, (**b**) CNC, and (**c**) TCNC.

Morphological studies on the synthesized TCNC were conducted using scanning electron microscope (SEM). In Figure 3, it is possible to observe the SEM image of TCNC (Figure 3c) compared to cellulose waste (Figure 3a) and CNC (Figure 3b).

From the SEM image in Figure 3a, it is possible to notice that the pristine cellulose shows high dimension agglomerates, while the hydrolysis process of cellulose using sulfuric acid greatly reduces its size and forms smaller agglomerates, making the cellulose more accessible and reactive (CNC, Figure 3b). Instead, after the transesterification reaction with sunflower oil, no significant differences in the morphology of CNC are observed, except for the formation of some small agglomerates, but always of limited dimensions (TCNC, Figure 3c).

**Figure 4:**
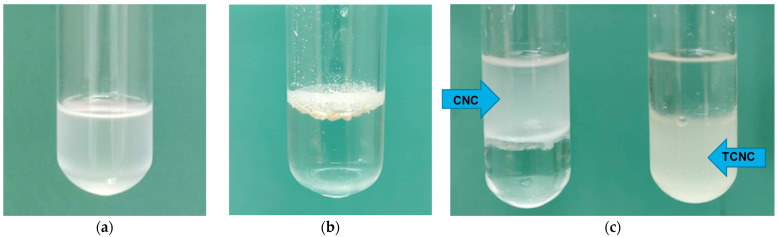
Hydrophobicity tests on (**a**) TCNC in CHCl_3_, (**b**) TCNC in water, and (**c**) CNC and TCNC in a biphasic system H_2_O/CHCl_3_.

Hydrophobicity tests were performed on TCNC and CNC, confirming that the hydrophobicity of TCNC was increased, rather than neat CNC (Figure 4).

In fact, as you can see, TCNC is well-dispersed in an organic solvent such as chloroform (Figure 4a), and, meanwhile, stays on the surface of the water (Figure 4b). Furthermore, in a biphasic system of water/chloroform (Figure 4c), the neat CNC, used for comparison, remains in the water phase, and, instead, the TCNC stays in the organic ones. These data confirm that the introduction of long alkyl chains on the surface of cellulose makes it more hydrophobic and, thus, more dispersible in biopolymers.

### 3.4. Preparation of Composite Films with PLA and Mechanical Tests

**Figure 5:**
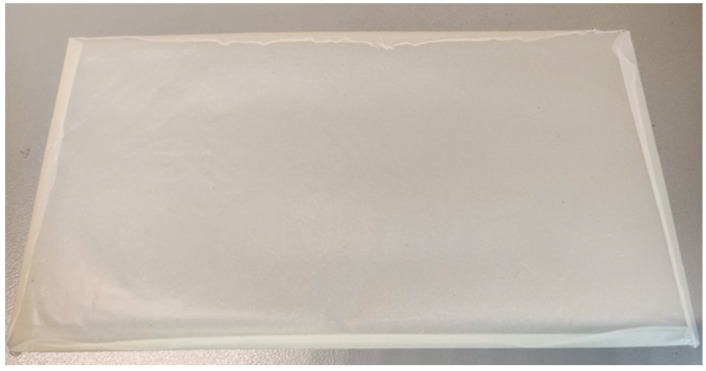
Representative composite film of PLA and TCNC.

Transesterified cellulose nanocrystals (TCNCs) were employed as an additive in the preparation of composite materials with the commercial polymer PLA, preparing several films at different percentages (1%, 3%, 5%, and 10%) and with a thickness ranging between 20–40 µm, by using the solvent casting method. For comparison, films with PLA and unmodified CNC were realized at the same percentages. A representative composite film obtained with PLA and TCNC as an additive is reported in Figure 5.

**Figure 6:**
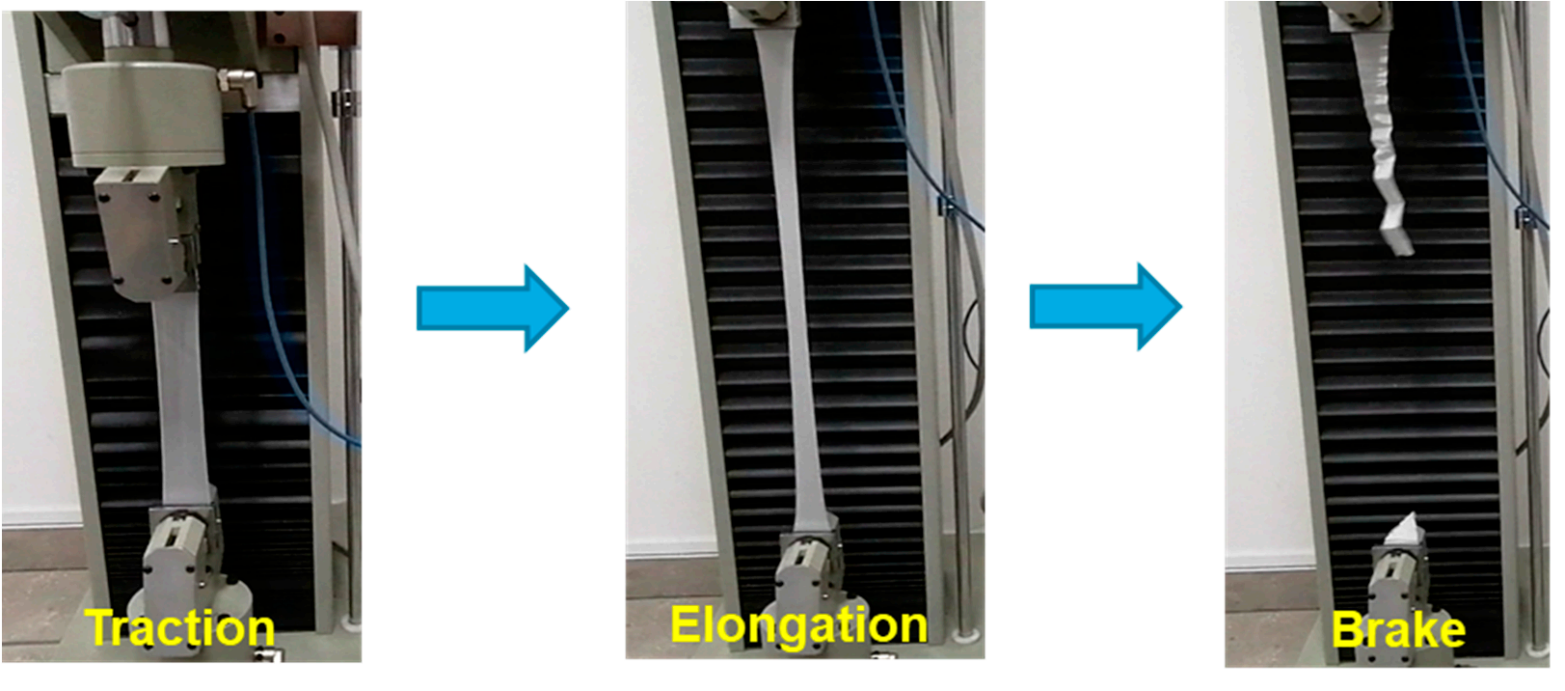
Sequence of mechanical tests for the prepared composite films.

The films were cut into appropriate shapes, and the mechanical properties of the composite materials were analyzed. In Figure 6, the schematic sequence of mechanical tests for the various prepared materials is reported.

**Figure 7:**
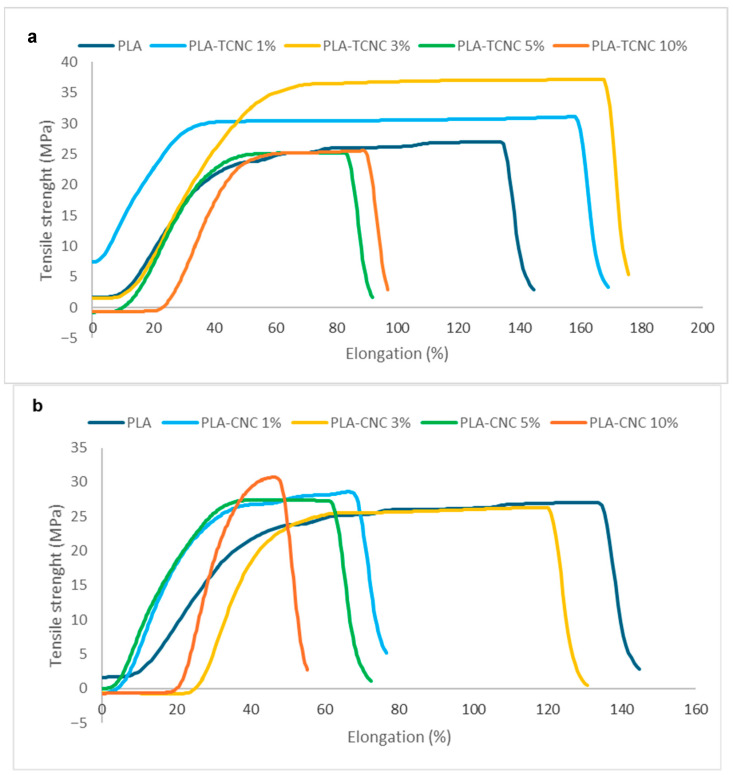
Stress–strain curves of the prepared composite materials in comparison to PLA film: (**a**) PLA-TCNC, and (**b**) PLA-CNC.

In Figure 7, the graphics of the tensile strength (σ) versus elongation at break (ε_b_) for all prepared composite films are reported.

As you can see from the data reported in Figure 7a, the best result was obtained for the load supported (σ) and elongation at break (ε_b_) when TCNC is used as an additive at 3% (yellow line), even if a significant increase was noted also for the addition of TCNC at 1% (light blue line). Instead, in the presence of higher concentrations of the additive, such as 5% and 10% (green and red lines, respectively), there was a decrease in all the mechanical properties of the obtained composite materials, because of an excess of TCNC, which can probably generate local breaking points with a consequent reduction in the performance of the final materials. Alternatively, the addition of neat CNC to PLA at the same percentages (1%, 3%, 5%, and 10%, Figure 7b) furnished composite materials that, in all cases, showed an increase in the load with respect to pure PLA (blue line), in agreement with the literature [35]. Despite this increase in load, both the elongation and the stiffness of the material were negatively affected by this additive.

**Figure 8:**
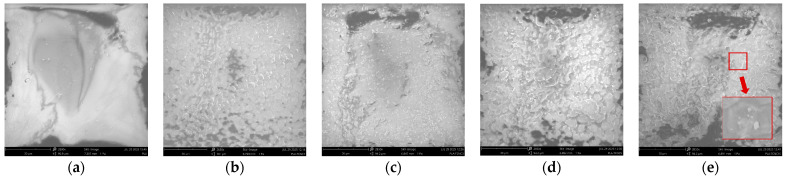
SEM of PLA film (**a**) and composite films of PLA-TCNC 1% (**b**), PLA-TCNC 3% (**c**), PLA-TCNC 5% (**d**), and PLA-TCNC 10% (**e**).

On the other hand, the SEM analysis (Figure 8) of composite films of PLA-TCNC at 1% and 3% showed a more homogeneous dispersion and higher miscibility of TCNC in PLA with respect to the case of the 5% and, especially, 10% composite films, in which agglomerates are present on the surface of the material (see red box, Figure 8e) (see Supplementary Materials for original SEM images). In particular, in some preparations of PLA-TCNC 10% composite films, we have noted the presence of visible granules also perceptible to the touch.

**Figure 9:**
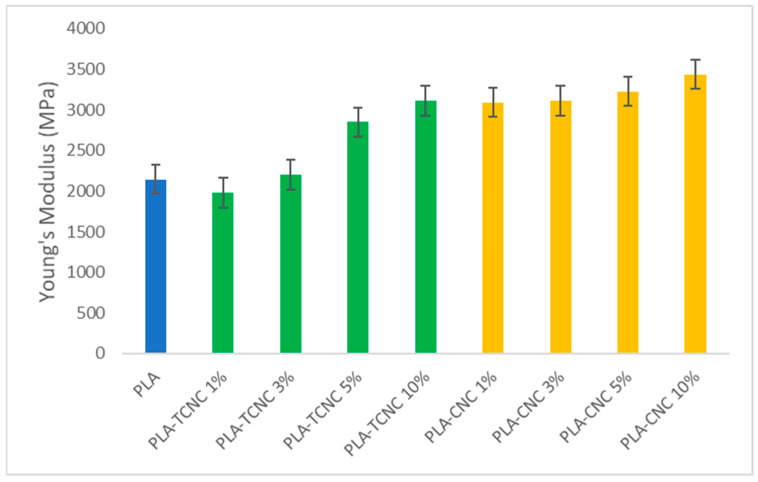
Young’s modulus of PLA film and the prepared composite films.

The elastic moduli of the composite films were calculated from their stress–strain curves and compared to that of the PLA film (Figure 9).

The composite films at 1% and 3% of TCNC as an additive (green) showed moduli similar to that of PLA (blue), whereas the Young’s modulus values increased for PLA-TCNC at 5% and 10% and for all composite materials with PLA-CNC (yellow), demonstrating a major stiffness for these composites.

### 3.5. Determination of Water Vapor Permeability Rate (WVTR)

**Figure 10:**
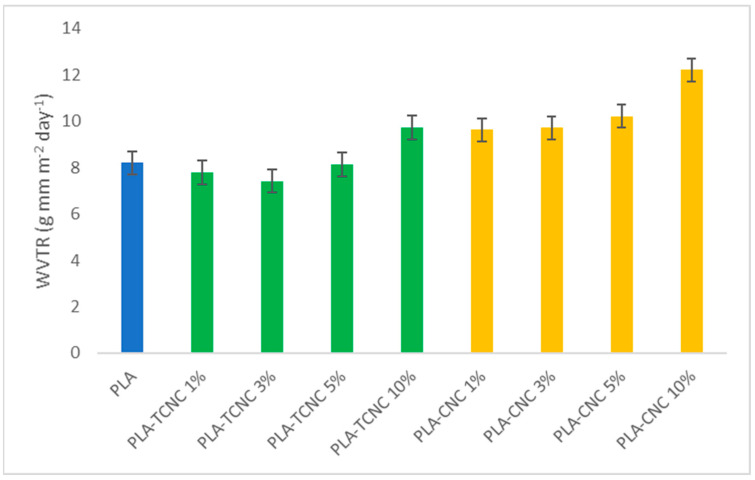
Water Vapor Transmission Rate (WVTR) on PLA film (blue), PLA-TCNC films at several percentages (green), and PLA-CNC films at several percentages (yellow).

The Water Vapor Transmission Rate (WVTR) can be used to determine how well a material composite resists moisture. We report the results measured for all composite films in Figure 10.

The barrier properties of a homogeneous two-component polymeric material are determined by both of its constituents. In fact, the increase in surface hydrophobicity is an efficient strategy with which to improve the barrier properties. In our case, the addition of a small amount of TCNC (i.e., 1% and 3%) to PLA renders the final composite films less hydrophilic than PLA, due to the hydrophobic effect of the alkyl-chain-based coating. On the contrary, an increased percentage of the additive (i.e., 5% and 10%) results in the more hydrophilic action of the OH groups being present on the additive dominant. For this same reason, the WVTR values observed for CNC as an additive in all considered percentages (1–10%) become higher than neat PLA.

### 3.6. Water Contact Angle (WCA) Measurements

**Figure 11:**
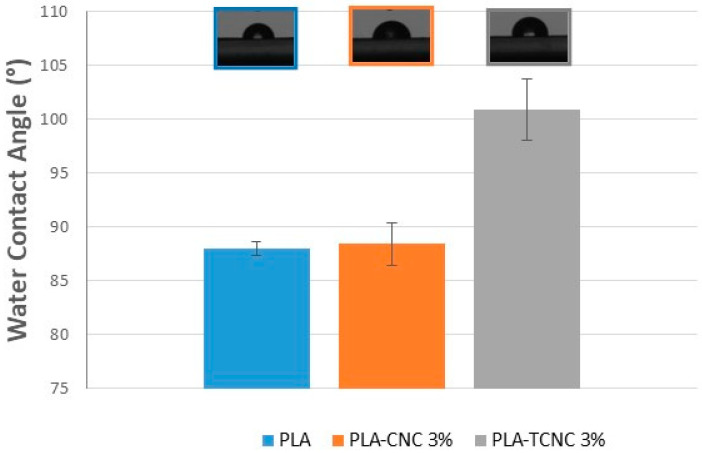
Water contact angle of the pristine PLA film (blue), and the composite films of PLA with CNC at 3% (orange) or TCNC at 3% (grey). At the top of the figure is reported a picture of a water drop deposited on a composite material surface.

Contact angle measurement is a common technique used to assess the surface wettability and hydrophobicity/hydrophilicity of materials. In Figure 11, we report the WCA measurements of pristine PLA film (blue), PLA-CNC film at 3% (orange), and PLA-TCNC film at 3% (grey).

As it is possible to observe, the film of pure PLA has a high-water wettability (WCA < 90°). The addition of 3% of CNC does not change surface wettability in a relevant way. On the contrary, the addition of 3% of TCNC to PLA confers hydrophobic properties to the composite film surface (WCA > 90 °C). In fact, the PLA-TCNC at 3% shows a 14.7% increase in WCA with respect to the pristine PLA film. The results obtained demonstrate the possibility to tailor the film–liquid affinity by an appropriate functionalization of the CNC used as an additive (at a low content) in the composite material.

## 4. Conclusions

With the aim of exploring sustainable strategies for the recovery and valorization of waste materials, in this work, we realized composite biofilms, starting from a natural biopolymer (PLA) reinforced with functionalized nanocellulose. By adhering to the principles of green chemistry, our work sought to breathe new life into industrial by-products, transforming them into innovative materials with promising applications in technology and industry. This approach aligns with global efforts to reduce waste, lower environmental impacts, and foster a circular economy.

Our research started from the development of a method to synthesize cellulose nanocrystals (CNCs) through sulfuric acid hydrolysis, starting from cellulose derived from orange peel waste (OPW). Successively, we performed surface modifications of cellulose nanocrystals, realizing a novel methodology of the transesterification reaction between CNC and exhausted sunflower oil enriched with oleic acid (HOSO). This reaction was carried out using waste reagents and tartaric acid as a catalyst under relatively mild conditions (i.e., the absence of a solvent, open-system reaction conditions, relatively low temperatures, and reduced reaction time), achieving a high percentage of grafting for the final product. In this contest, considering the novelty of the reaction, we proposed a mechanism of the transesterification reaction between CNC and triglycerides of HOSO catalyzed by tartaric acid.

The resulting transesterified CNC (TCNC) was characterized and utilized as an additive in composite films of PLA at different percentages. The best performances were achieved, especially when TCNC was added at a concentration of 3%, showing better hydrophobicity, with an improvement in the mechanical and barrier properties of the final film. Therefore, the obtained results demonstrate that waste such as cellulose and exhausted sunflower oil can be modified and integrated into advanced materials to address the current environmental and industrial challenges. In fact, in the context of sustainability, the entire process has an excellent environmental impact, transforming waste materials into bio-composite materials with high added value.

## Figures and Tables

**Table 1:** Reaction condition optimization of the transesterification reaction between exhausted sunflower oil and nanocellulose.

Entry	Acid	Temperature (°C)	Time (h)	Grafting (%)
1	Tartaric	120	4	32
2	Tartaric	120	6	47
3	Tartaric	120	8	48
4	Tartaric	120	overnight	50
5	Tartaric	140	6	43
6	Tartaric	160	6	38
7	Malic	120	6	33
8	Succinic	120	6	21
9	Adipic	120	6	-
10	Oxalic	120	6	-
11	-	120	6	-

## Data Availability

The original contributions presented in this study are included in the article/Supplementary Materials. Further inquiries can be directed to the corresponding authors.

## Supplementary Materials

The following supporting information can be downloaded at: https://www.mdpi.com/article/10.3390/polym17212877/s1, Figure S1: FT-IR of CNC (**1**); Figure S2: FT-IR of exhausted sunflower oil HOSO (**2**); Figure S3: FT-IR of TCNC (**3**); Figure S4: FT-IR of diacetyl tartaric acid (**7**); Figure S5: FT-IR of acetyl CNC (**8**); Figure S6: ^1^H NMR of TCNC (**3**); Figure S7: ^1^H NMR of diacetyl tartaric acid (**7**); Figure S8: COSY NMR of diacetyl tartaric acid (**7**); Figure S9: ^13^C NMR of diacetyl tartaric acid (**7**); Figure S10–S17: SEM images of waste cellulose, CNC, TCNC, PLA, PLA-TCNC1%, PLA-TCNC3%, PLA-TCNC5%, and PLA-TCNC10%; and Figure S18: WVTR measurements.
