# Supplementary material for: Catalytic Transesterification of Cellulose Nanocrystals (CNCs) with Waste Oils: A Sustainable and Efficient Route to Form Reinforced Biofilms
Source: Polymers (Basel). 2025 Oct 28;17(21):2877. doi: 10.3390/polym17212877 (PMC12609332; doi:10.3390/polym17212877)
Supplement: Supplementary file 1 [file polymers-17-02877-s001.zip › polymers-3908105-supplementary.pdf]

Supplementary Materials

Catalytic Transesterification of Cellulose Nanocrystals (CNCs) with Waste Oils: A Sustainable and Efficient Route to Form Reinforced Biofilms

Antonio De Nino [1], Antonio Jiritano [1,*], Federica Meringolo [1], Paola Costanzo [1], Vincenzo Algieri [2], Enrica Fontananova [3] and Loredana Maiuolo [1,*]

[1] Department of Chemistry and Chemical Technologies, University of Calabria, Via P. Bucci, Cubo 12C,
87036 Rende, CS, Italy; denino@unical.it (A.D.N.); federica.meringolo@unical.it (F.M.); paola.costanzo@unical.it (P.C.)

[2] IRCCS NEUROMED—Istituto Neurologico Mediterraneo, Via Atinense 18, 86077 Pozzilli, IS, Italy; vincenzo.algieri@unical.it (V.A.)

[3] Institute on Membrane Technology of the National Research Council (ITM-CNR), University of Calabria, Via P. Bucci, Cubo 17/C, 87036 Rende, CS, Italy; e.fontananova@itm.cnr.it

* Correspondence: antonio.jiritano@unical.it (A.J.); maiuolo@unical.it (L.M.)

FT-IR Spectra

Cellulose nanocrystals (CNC) (**1**)

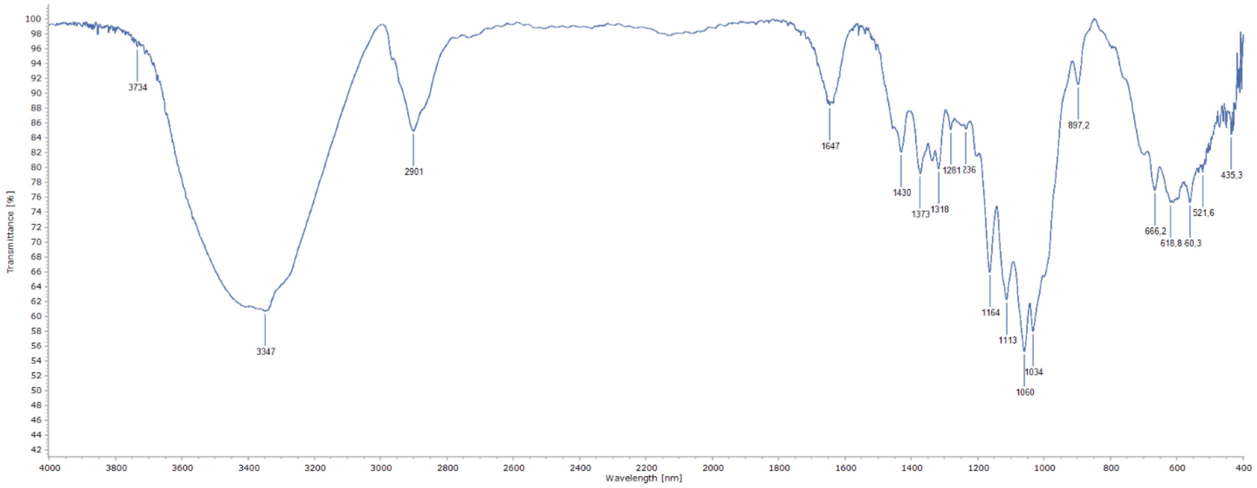

Figure S1

Exhausted sunflower oil HOSO (**2**)

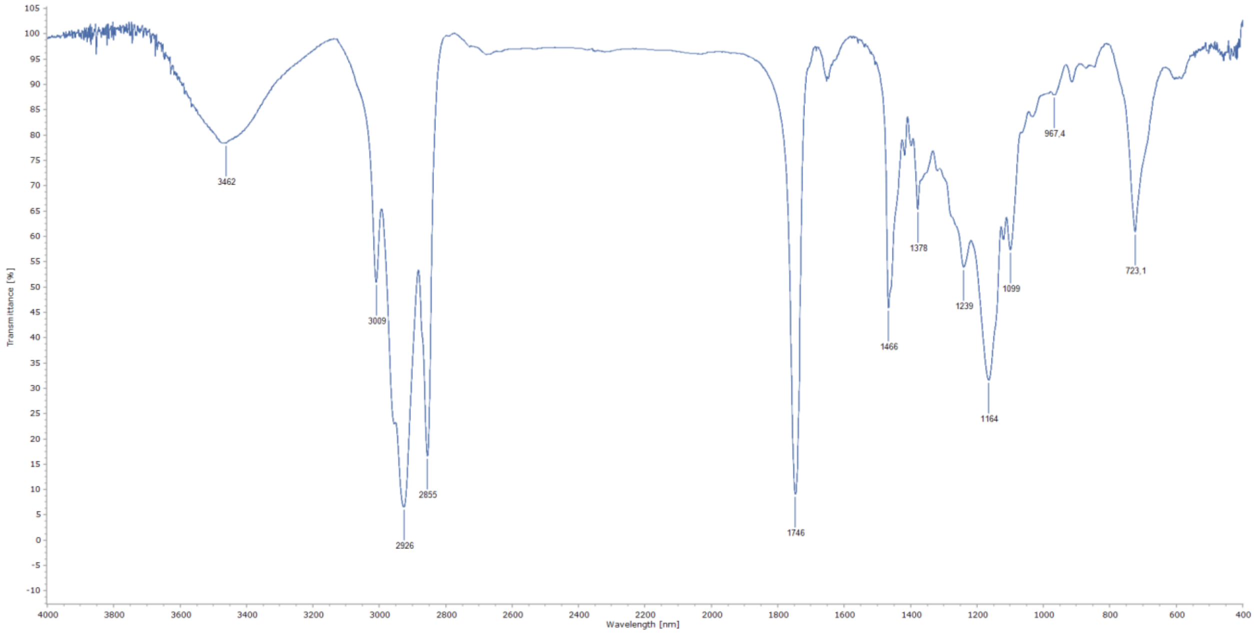

Figure S2

Transesterified CNC (**3**)

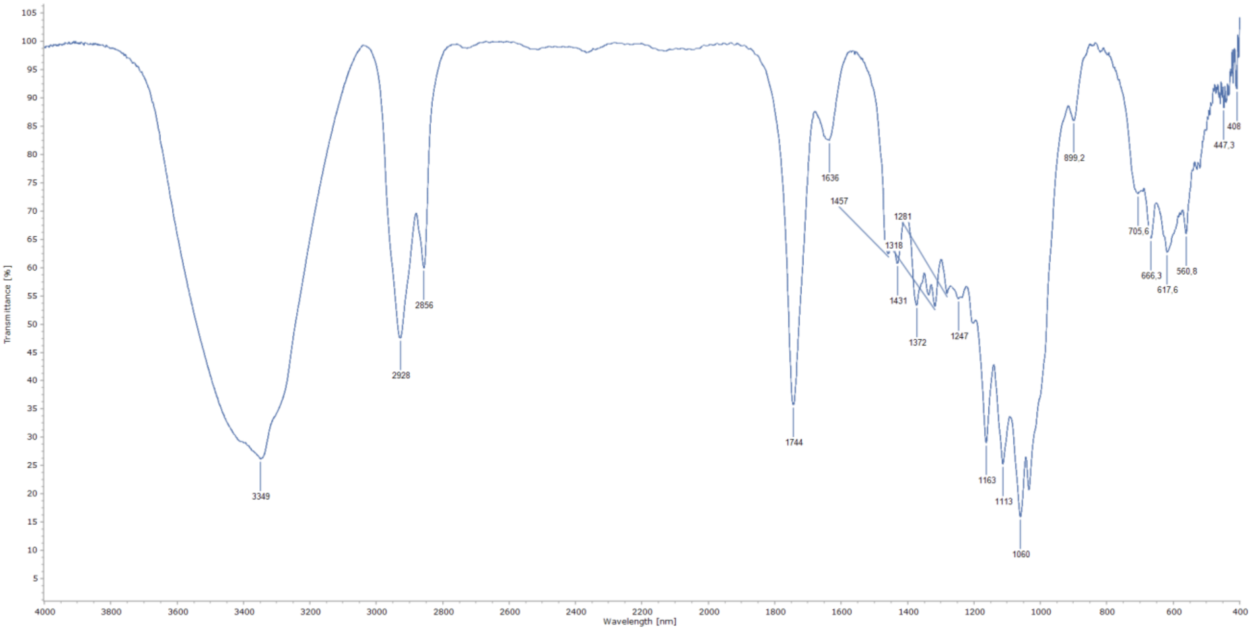

Figure S3

Diacetyl tartaric acid (**7**)

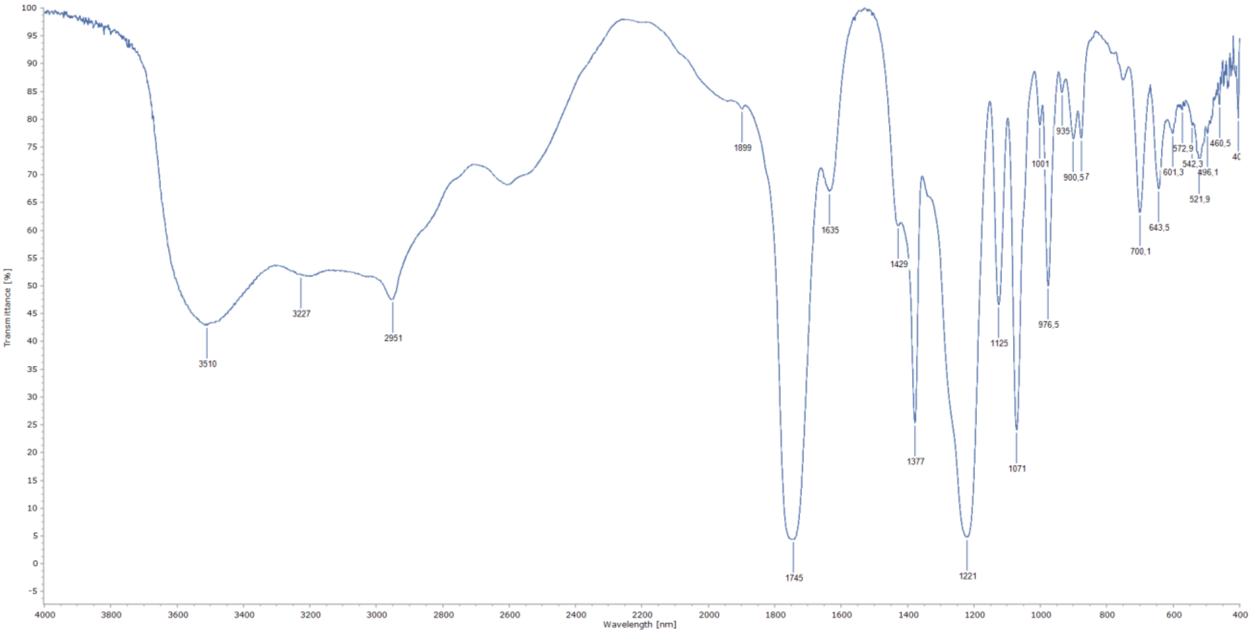

Figure S4

Acetyl CNC (**8**)

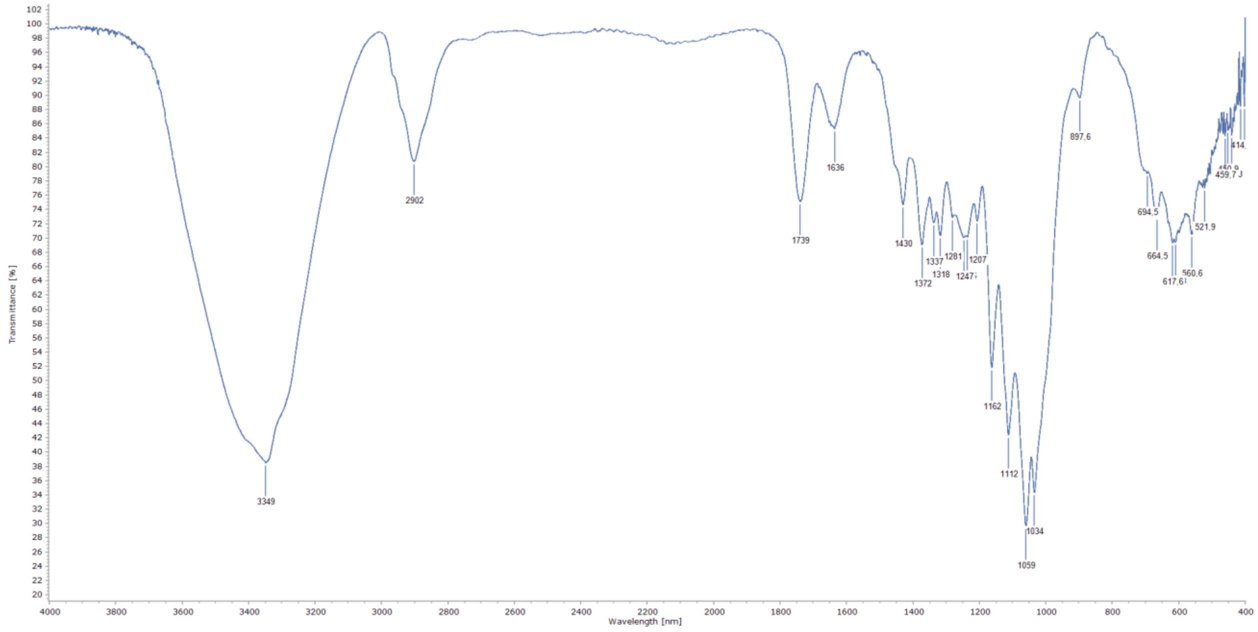

Figure S5

NMR spectrum of TCNC (**3**)

^{1}H NMR (500 MHz, DMSO-*d_6*, 1024 scans, T = 37 °C)

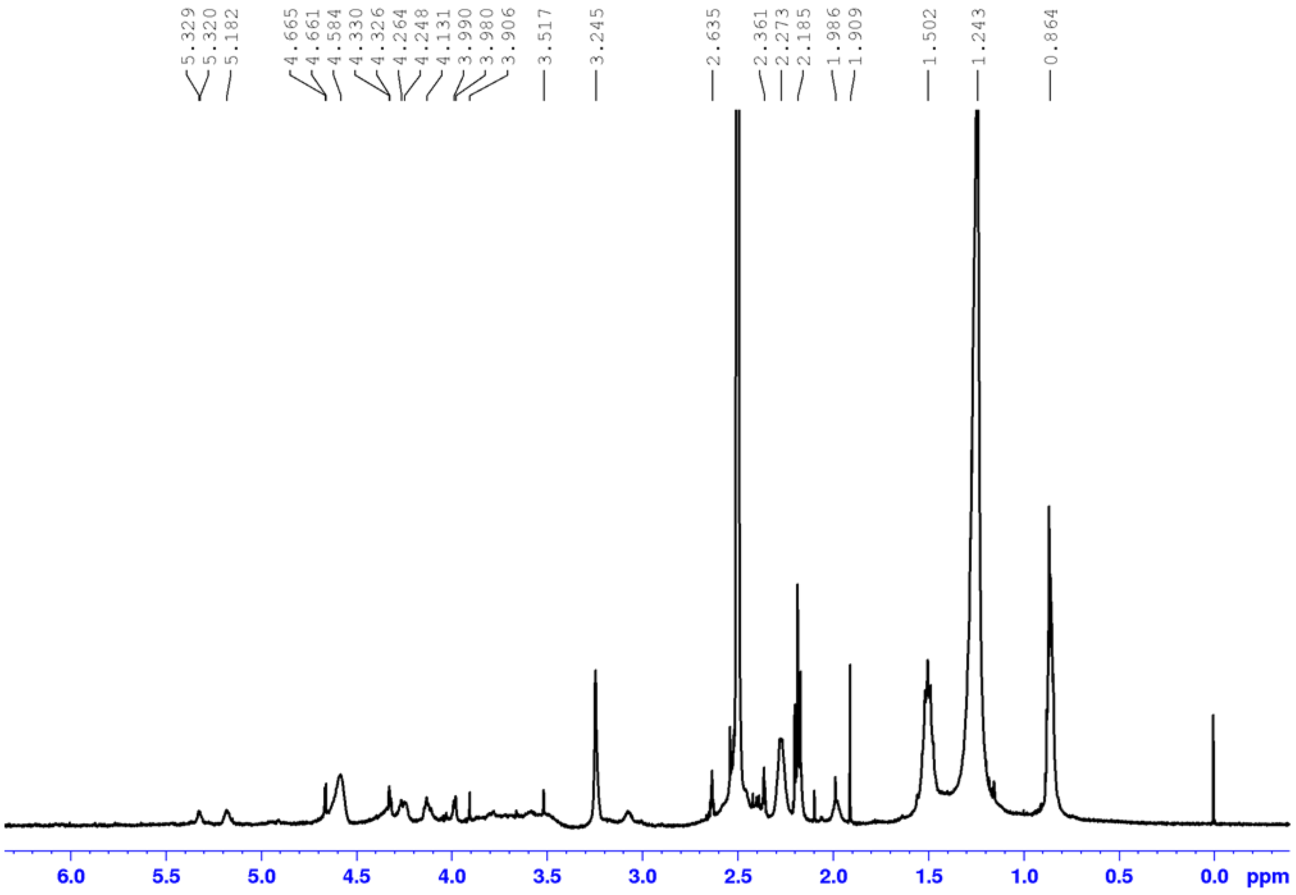

Figure S6

NMR spectra of diacetyl tartaric acid (**7**)

^{1}H NMR (500 MHz)

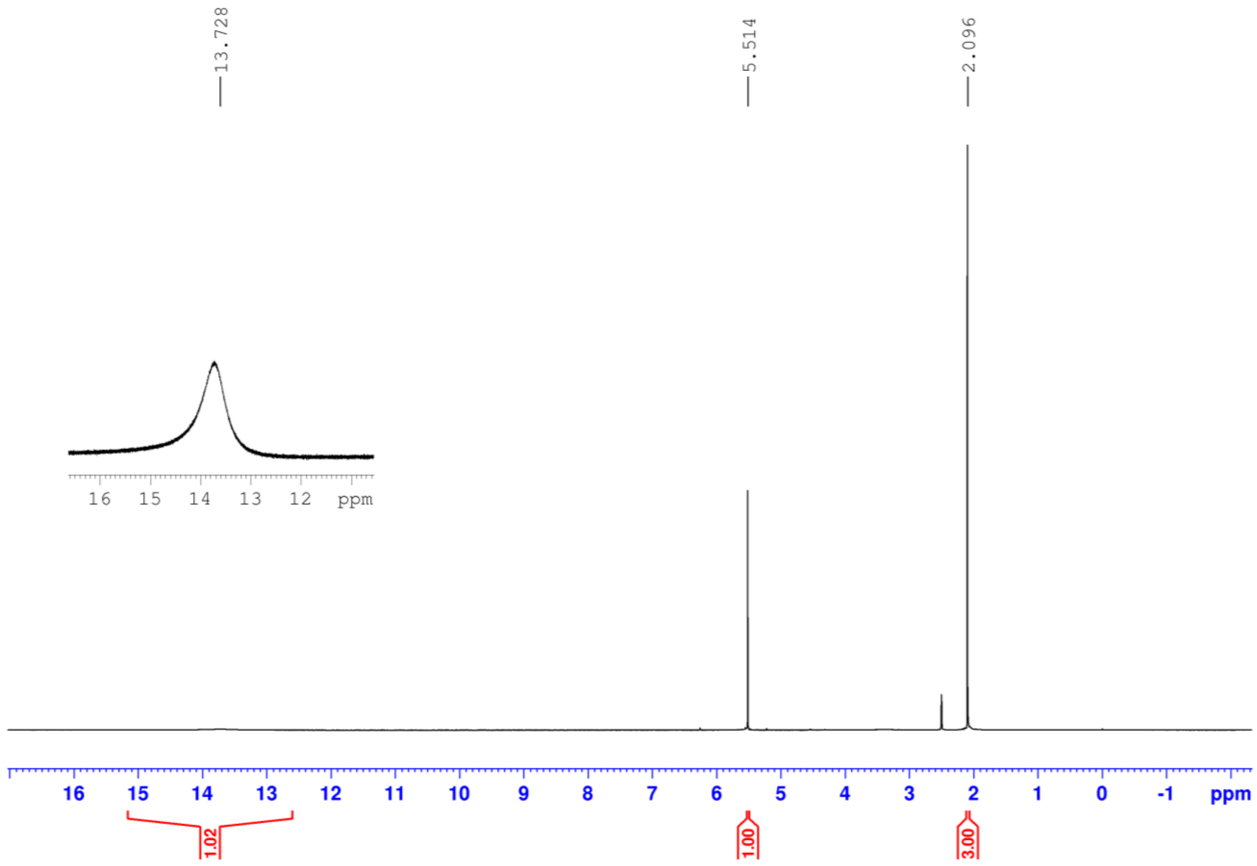

Figure S7

COSY NMR (500 MHz)

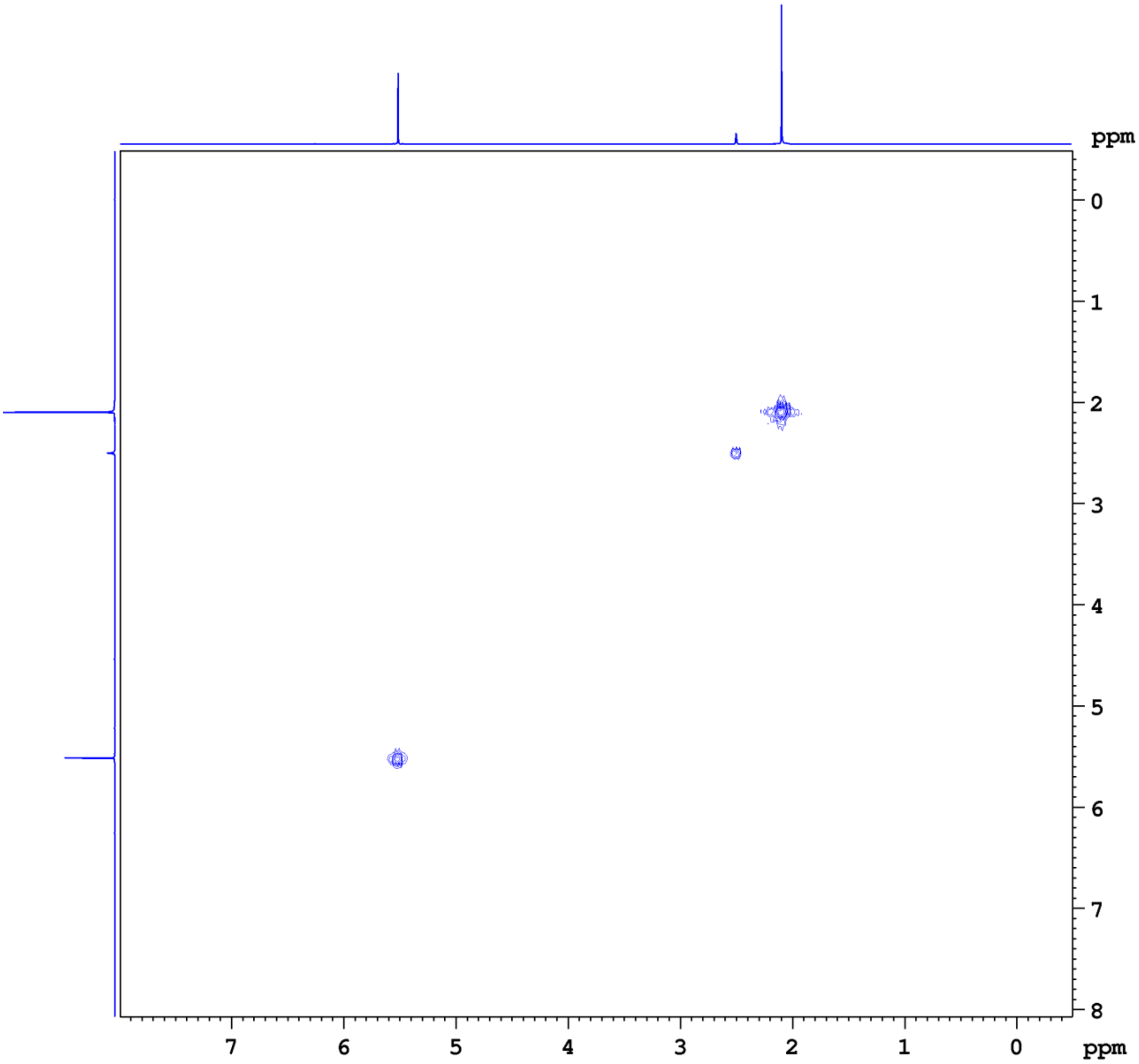

Figure S8

^{13}C NMR (125 MHz)

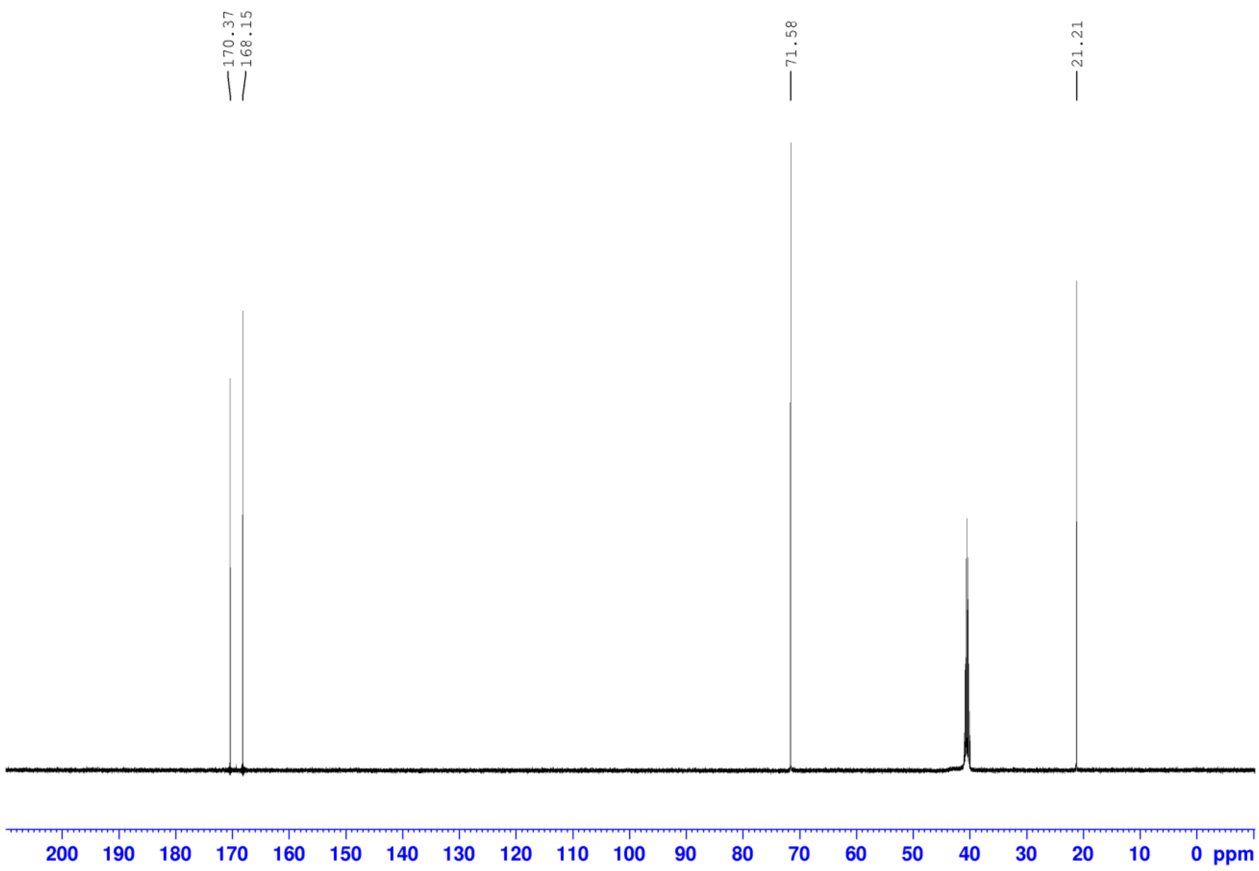

Figure S9

SEM images

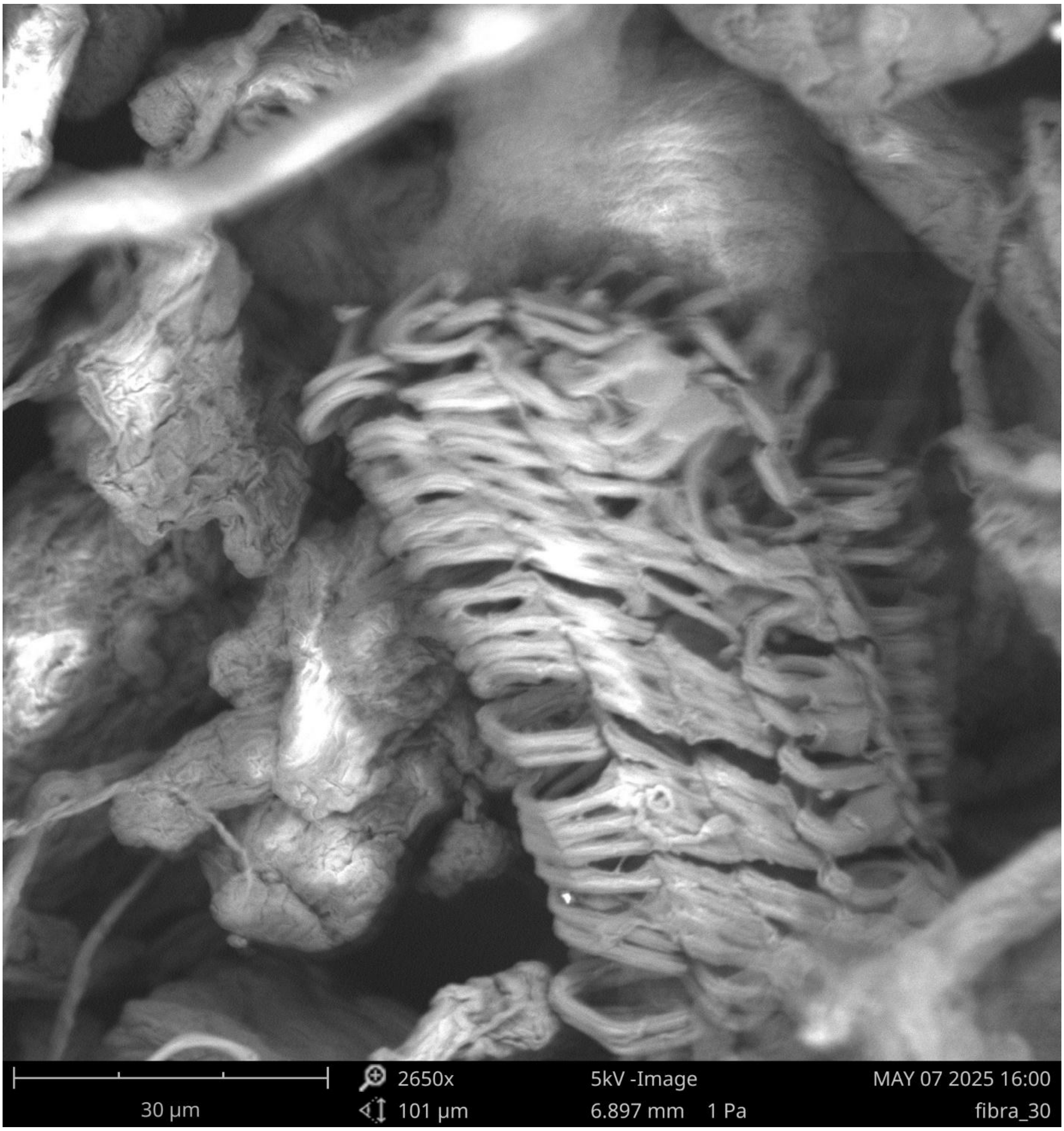

Figure S10 - Cellulose waste deriving from orange peels

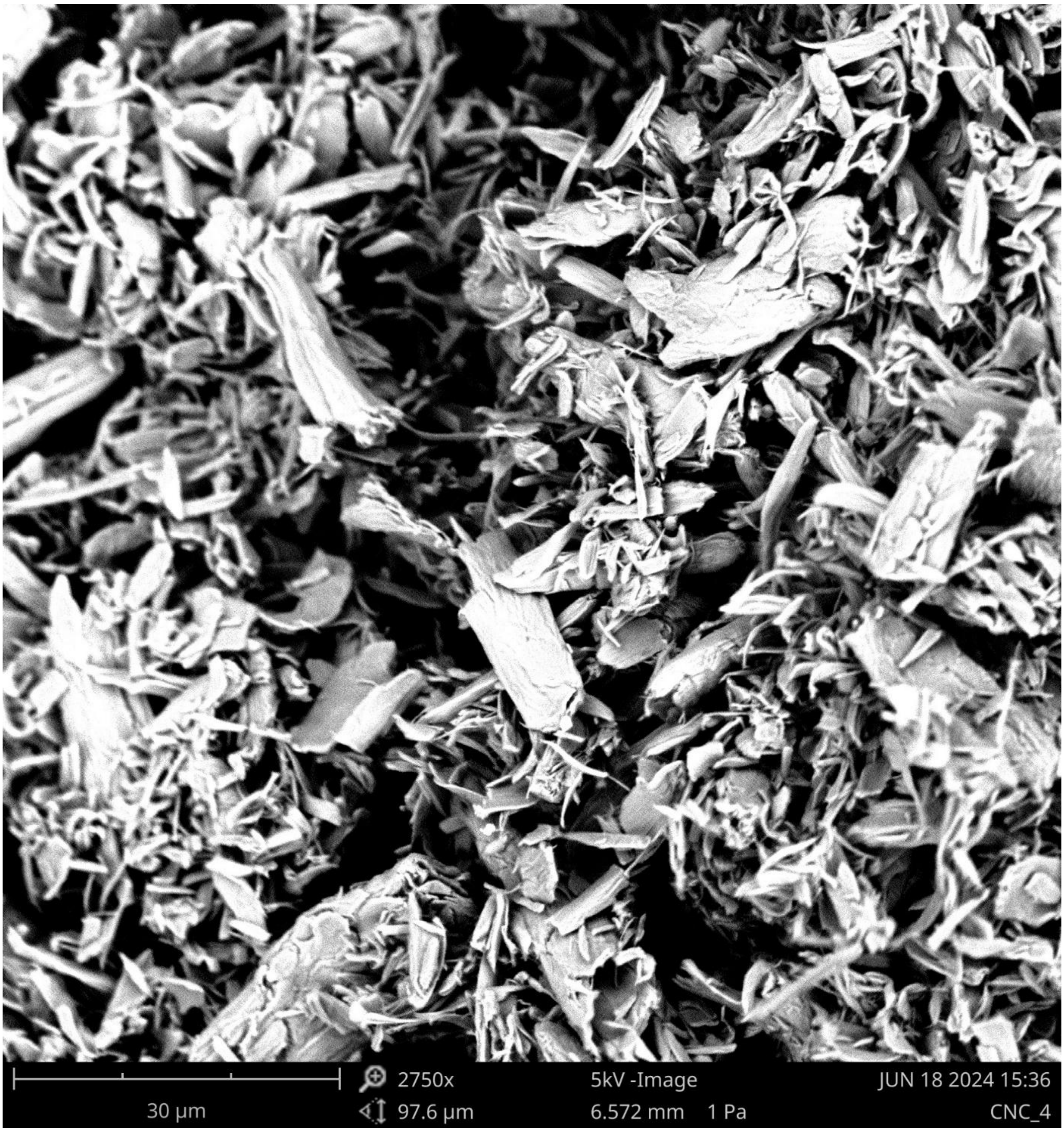

Figure S11 - CNC

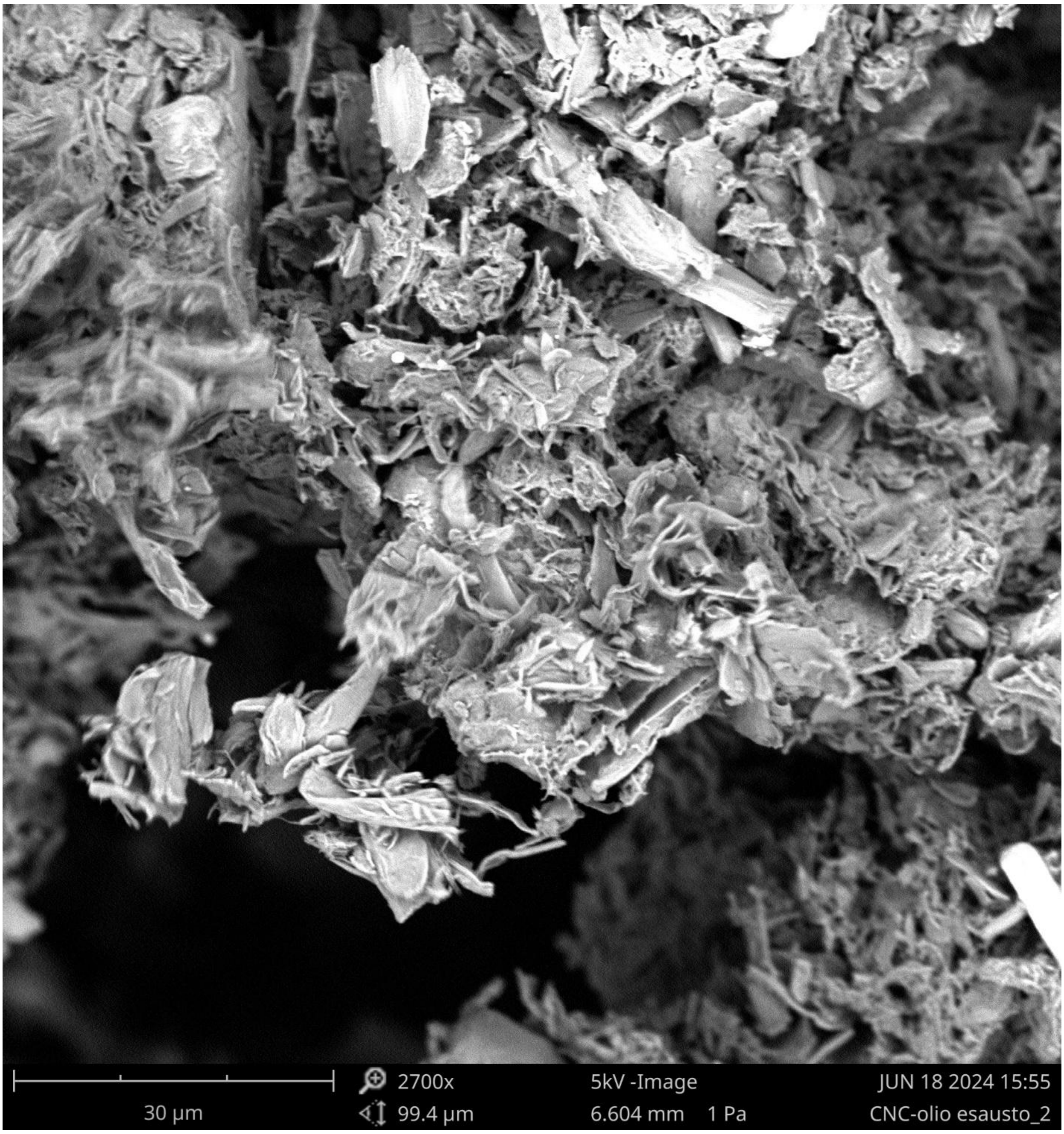

Figure S12 - TCNC

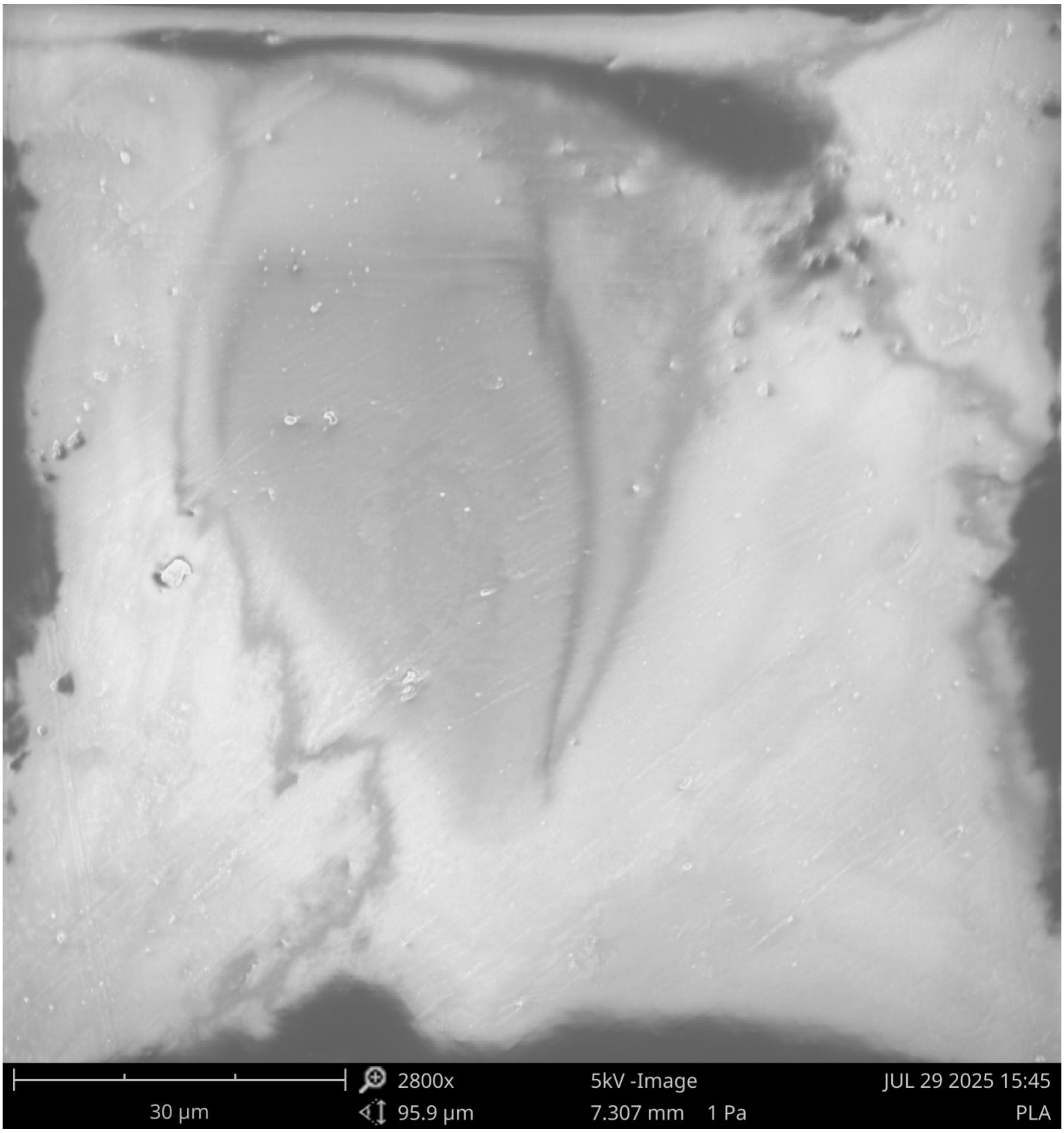

Figure S13 - PLA

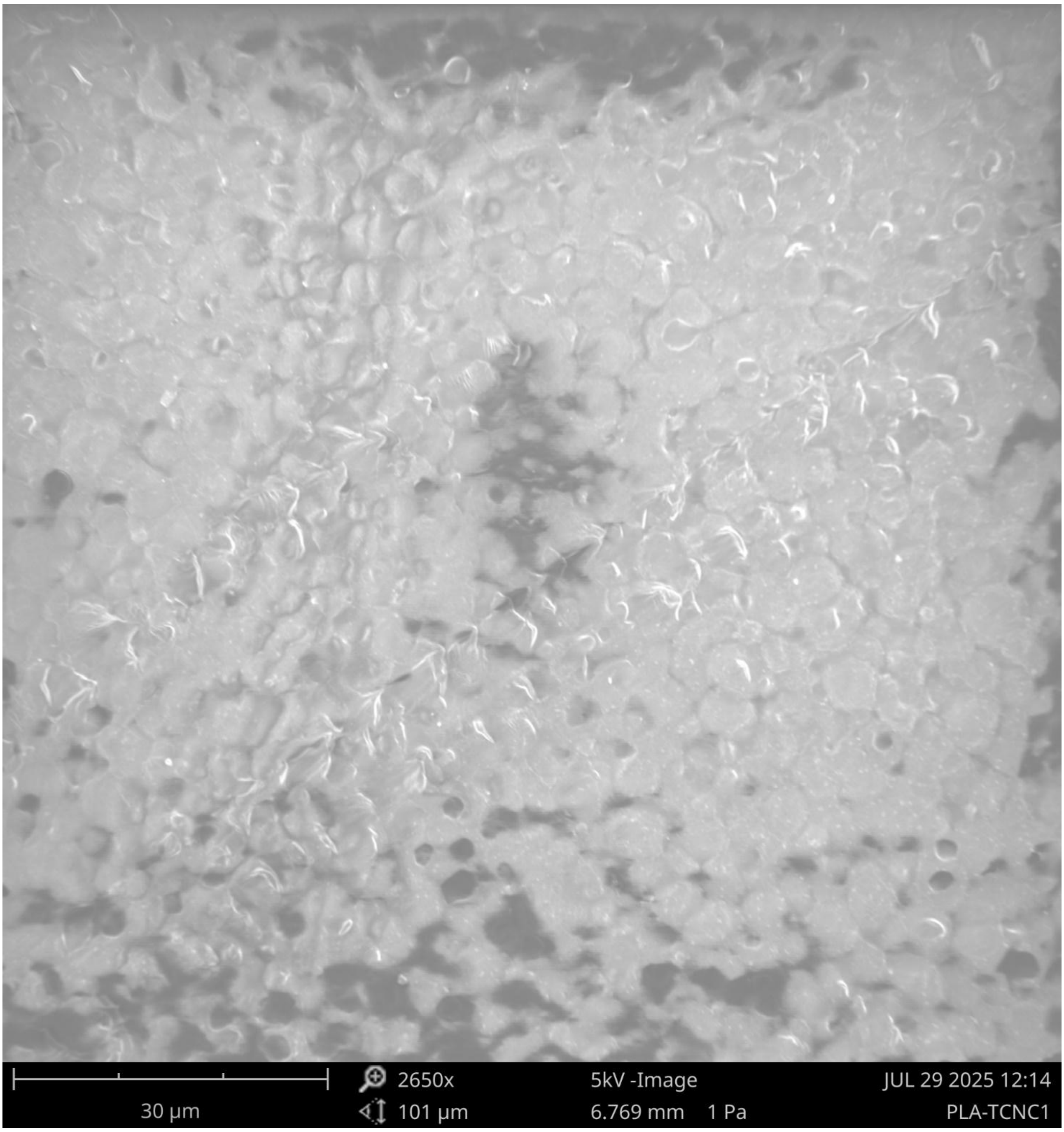

Figure S14 - PLA-TCNC 1%

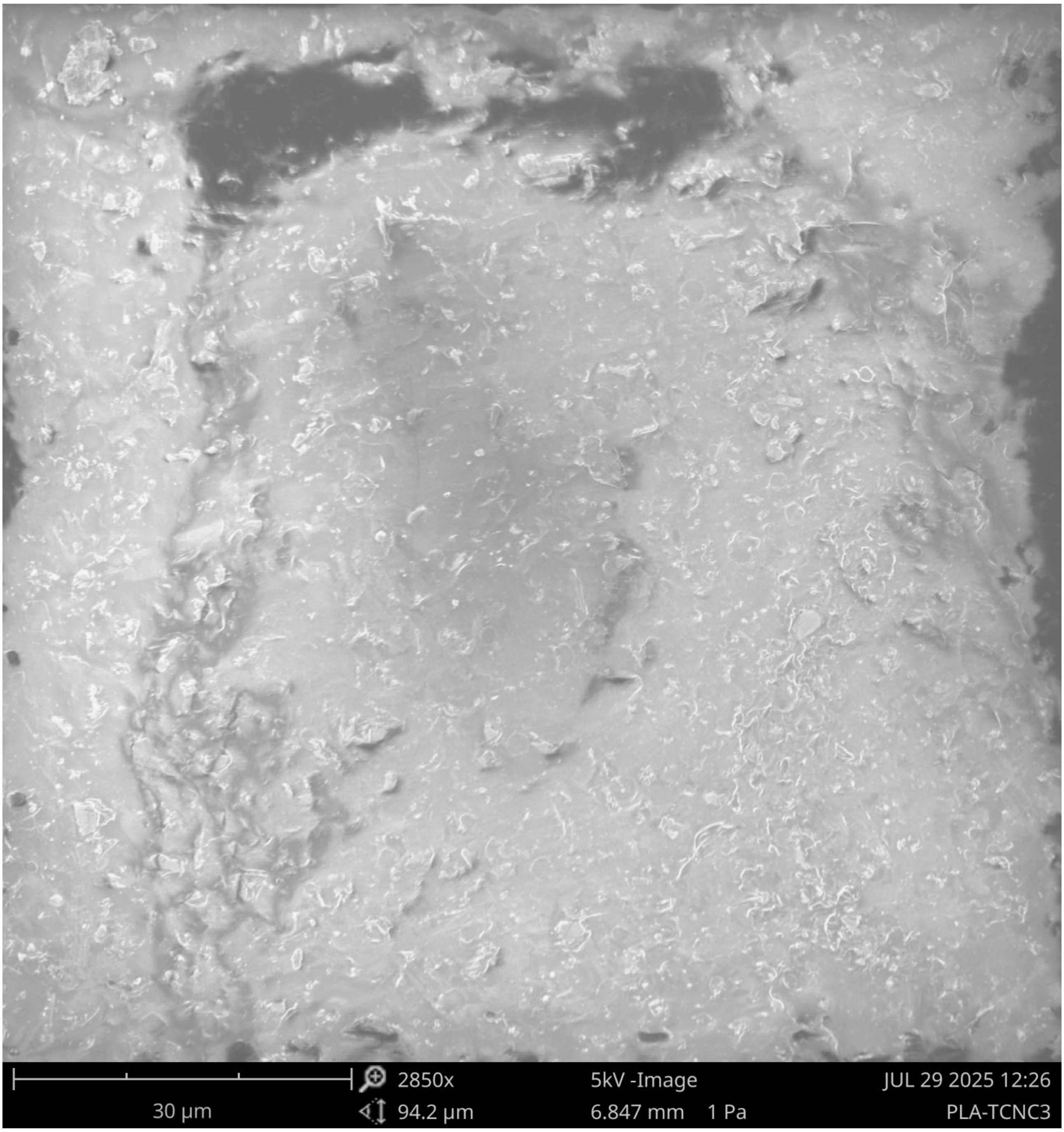

Figure S15 - PLA-TCNC 3%

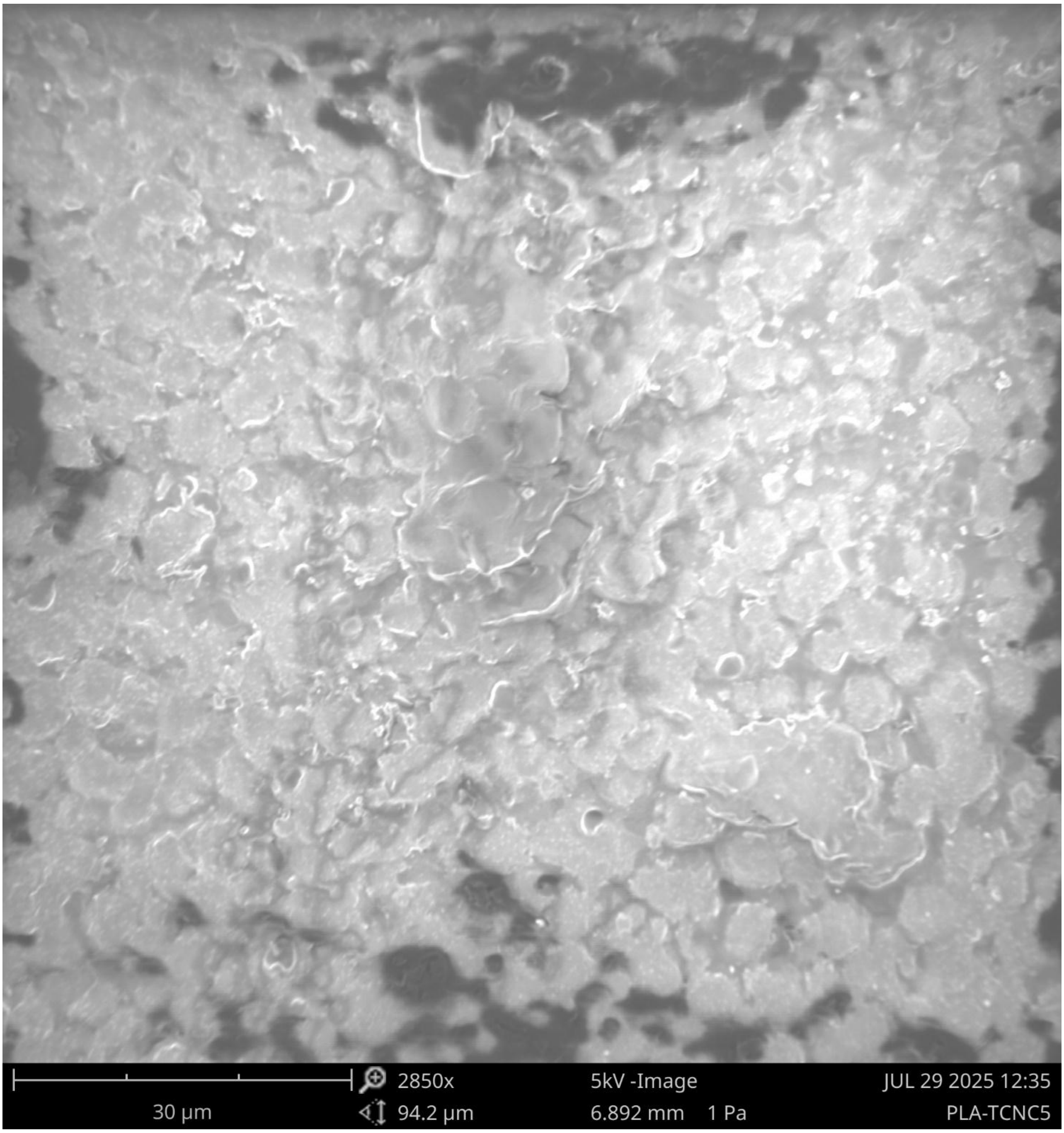

Figure S16 - PLA-TCNC 5%

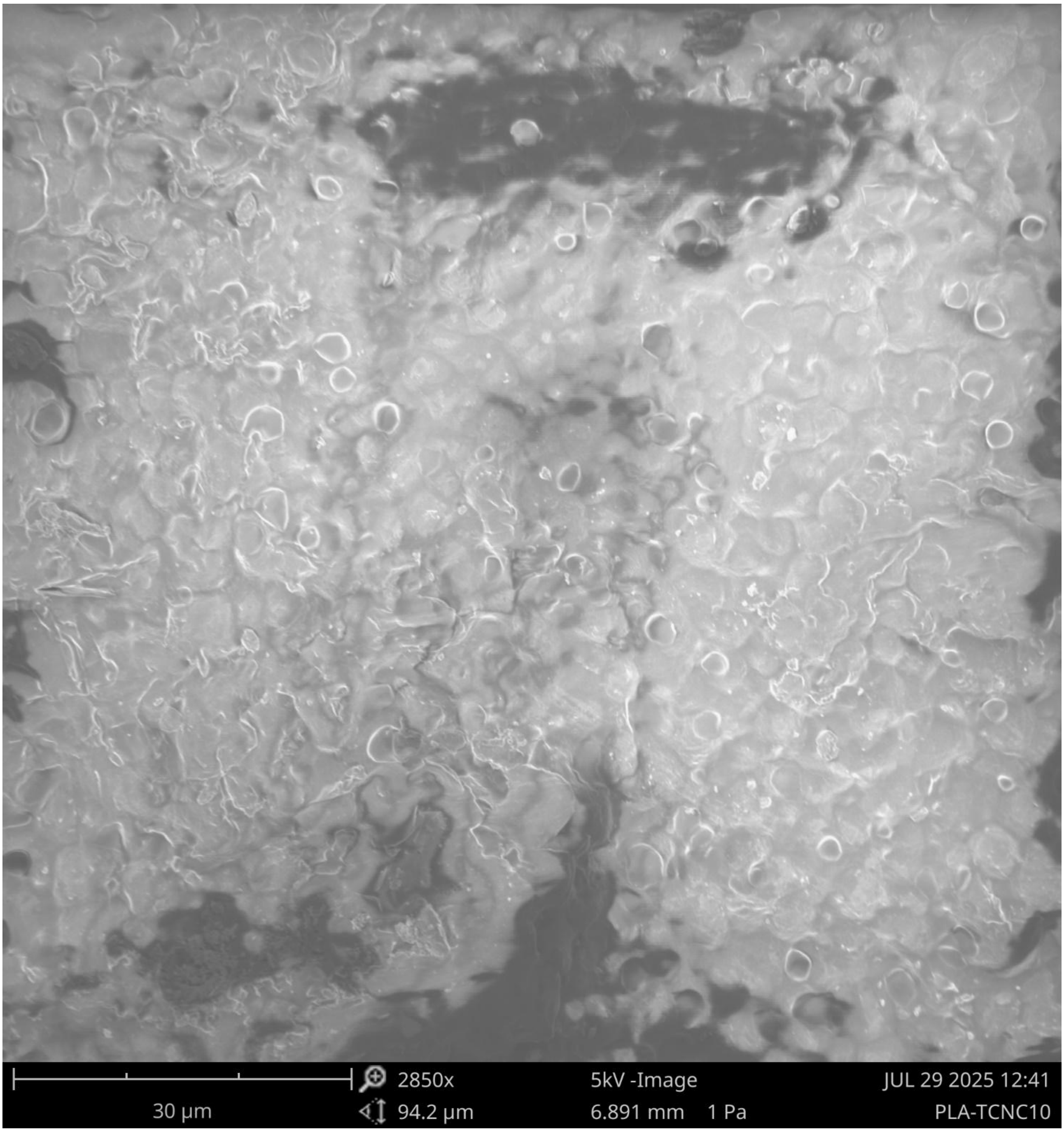

Figure S17 – PLA-TCNC 10%

WVTR measurements

	1	2	3	stdv
PLA	8.21	8.32	8.18	0.073711
PLA-TCNC 1%	7.79	7.71	7.82	0.056862
PLA-TCNC 3%	7.42	7.39	7.51	0.06245
PLA-TCNC 5%	8.14	8.21	8.1	0.055678
PLA-TCNC 10%	9.73	9.69	9.75	0.030551
PLA-CNC 1%	9.63	9.71	9.58	0.065574
PLA-CNC 3%	9.71	9.87	9.7	0.095394
PLA-CNC 5%	10.22	10.31	10.13	0.09
PLA-CNC 10%	12.21	12.08	12.29	0.105987

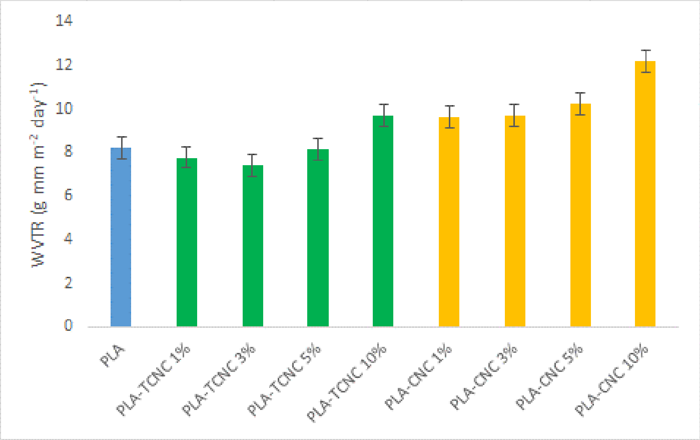

Figure S18
